# Osteopontin That Is Elevated in the Airways during COPD Impairs the Antibacterial Activity of Common Innate Antibiotics

**DOI:** 10.1371/journal.pone.0146192

**Published:** 2016-01-05

**Authors:** Anele Gela, Ravi K. V. Bhongir, Michiko Mori, Paul Keenan, Matthias Mörgelin, Jonas S. Erjefält, Heiko Herwald, Arne Egesten, Gopinath Kasetty

**Affiliations:** 1 Respiratory Medicine & Allergology, Department of Clinical Sciences Lund, Lund University, SE-221 84, Lund, Sweden; 2 Infection Medicine, Department of Clinical Sciences Lund, Lund University, SE-221 84, Lund, Sweden; 3 Airway Inflammation Unit, Department of Experimental Medical Sciences, Lund University, SE-221 84, Lund, Sweden; University of Tübingen, GERMANY

## Abstract

Bacterial infections of the respiratory tract contribute to exacerbations and disease progression in chronic obstructive pulmonary disease (COPD). There is also an increased risk of invasive pneumococcal disease in COPD. The underlying mechanisms are not fully understood but include impaired mucociliary clearance and structural remodeling of the airways. In addition, antimicrobial proteins that are constitutively expressed or induced during inflammatory conditions are an important part of the airway innate host defense. In the present study, we show that osteopontin (OPN), a multifunctional glycoprotein that is highly upregulated in the airways of COPD patients co-localizes with several antimicrobial proteins expressed in the airways. *In vitro*, OPN bound lactoferrin, secretory leukocyte peptidase inhibitor (SLPI), midkine, human beta defensin-3 (hBD-3), and thymic stromal lymphopoietin (TSLP) but showed low or no affinity for lysozyme and LL-37. Binding of OPN impaired the antibacterial activity against the important bacterial pathogens *Streptococcus pneumoniae* and *Pseudomonas aeruginosa*. Interestingly, OPN reduced lysozyme-induced killing of *S*. *pneumoniae*, a finding that could be explained by binding of OPN to the bacterial surface, thereby shielding the bacteria. A fragment of OPN generated by elastase of *P*. *aeruginosa* retained some inhibitory effect. Some antimicrobial proteins have additional functions. However, the muramidase-activity of lysozyme and the protease inhibitory function of SLPI were not affected by OPN. Taken together, OPN can contribute to the impairment of innate host defense by interfering with the function of antimicrobial proteins, thus increasing the vulnerability to acquire infections during COPD.

## Introduction

Chronic obstructive pulmonary disease (COPD) is a serious medical condition where both its prevalence and also mortality from the disease are increasing worldwide. COPD is characterized by a persistent inflammation that leads to loss of recoil resulting a decreased airflow, often in combination with emphysema and bronchiolitis [1]. Progression of the disease is strongly associated with the frequency of exacerbations [2]. Infections are an important cause of exacerbations where viruses and bacteria are estimated to play equal roles [3]. Bacteria of importance include non-typeable *Haemophilus influenzae* (Nt*Hi*), *Streptococcus pneumoniae*, *Branhamella catarrhalis*, and not least *Pseudomonas aeruginosa* [4, 5]. In addition, there is an increased risk of invasive pneumococcal disease (IPD) in COPD [6]. Despite the activation of innate immune mechanisms during the pathogenesis of COPD, which is evidenced by accumulation of inflammatory cells, chemokines and pro-inflammatory cytokines, infections trigger exacerbations in COPD [1]. This is likely due to a dysregulated and inappropriate host response [7, 8]. Antimicrobial proteins (AMPs) are important effector molecules of airway innate immunity [9]. As early as in 1922, Sir Alexander Fleming discovered lysozyme and demonstrated its antibacterial activity and also its presence in sputum [10]. Recent studies have demonstrated that many different AMPs such as lysozyme, lactoferrin, secretory leukocyte peptidase inhibitor (SLPI), midkine, defensins, thymic stromal lymphopietin (TSLP) and the cathelicidin-derived peptide LL-37 are expressed in human airways where airway epithelial cells, submucosal glands, and recruited neutrophils are important cellular sources [11–17]. Most AMPs are cationic with a net charge of (+2 to +9] and often adopt an amphipathic structure essential for their antimicrobial action, features that are important for their disrupting activities on the bacterial cell membrane [9].

Osteopontin (OPN) is an anionic, phosphorylated glycoprotein, expressed by many tissues and immune cells during a number of physiological and pathological processes [18]. OPN has been involved in the regulation of inflammation, acting as a chemotactic factor for T-cells, macrophages and neutrophils and modulating the function and differentiation of these inflammatory cells [18, 19]. Recent studies have demonstrated high levels of OPN in patients with COPD, where the levels increased with disease severity [20, 21]. Considering the association between high OPN expression and COPD, we investigated whether OPN impairs the activities of AMPs, thus explaining the vulnerability to acquire infections in diseases characterized by chronic airway inflammation as exemplified by COPD.

## Materials and Methods

### Ethics statement

All subjects gave their written informed consent to participate in the study, which was approved by the Regional Ethical Committee in Lund, Östra Vallgata 14, SE-223 61 Lund, Sweden.

### Reagents

Reagents were obtained as follows: human neutrophil lysozyme and lactoferrin purified from human milk (Sigma-Aldrich, St. Louis, MO), recombinant human (rh)SLPI (R&D Systems, Minneapolis, MI) rhOPN, hBD-3, midkine, and TSLP (Peprotech, London, UK). The peptides LL-37 and VSS60 were synthesized using fmoc-chemistry (Schafer-N, Copenhagen, Denmark). Rabbit anti-serum against OPN was generously provided by Dr. Dick Heinegård, Lund, Anti-lactoferrin antibody (Abcam, Cambridge, UK), Anti-human lysozyme (Dako, Glostrup, Denmark), and anti-human SLPI antibody (R&D Systems, Minneapolis, MI).

### Immunohistochemistry

COPD lung tissues were obtained from patients with COPD (GOLD stage IV) undergoing lung transplantation surgery at Skåne University Hospital, Lund, Sweden. The clinical characteristics of the patients are shown in S1 Table. At the time of surgery, the patients suffered neither from an ongoing exacerbation nor an infection. Distal tissue samples were submerged in 4% buffered paraformaldehyde. After dehydration and paraffin embedding, 3 μm thin parallel sections were generated from the tissue blocks. After rehydration and antigen retrieval, sections were incubated with antibodies against OPN, lactoferrin, lysozyme, and SLPI, and bound antibodies were detected using horseradish peroxidase-conjugated secondary antibodies and visualized using 3,3-diaminobenzidine as chromogen (K8010, Dako, Glostrup, Denmark). All staining procedures were performed in an automated slide-staining robot (DakoCytomation, Glostrup, Denmark).

### Surface plasmon resonance spectrometry

Binding characteristics between OPN and the AMPs investigated were carried out using a BIAcore X-100 instrument (GE Healthcare, Uppsala, Sweden). Human OPN was immobilized on a CM5 sensor chip (GE Healthcare) by standard amine coupling and the resonance units (RU) were calculated based on the analyte molecular weight. In parallel, one flow cell was incubated with buffer alone (i.e. without OPN), serving as control. Interaction experiments were performed with injections of 31.25, 62.5, 125, 250, 500 nM of AMPs in running buffer (10 mM HEPES, pH 7.5, 150 mM NaCl, 0.005% surfactant P20, and 3.4 mM EDTA) at a flow rate of 30 μl/min. After the end of each injection, dissociation was performed with 0.5 M NaCl for 10 min, followed by a washing procedure. After X and Y normalization of data, the blank curves from the control flow cell of each injected concentration were subtracted. The BIA evaluation 3.1 analysis software (GE Healthcare) was used to determine equilibrium dissociation constants (K_D_) from the processed data sets by fitting to a 1:1 molecular binding model with drifting baseline.

### ELISA-based OPN binding assay

A 96-well ELISA plate was coated with 0.25, 1, and 2 μg of AMPs lactoferrin, lysozyme, SLPI, midkine, hBD3, LL37 or TSLP and incubated at 4°C over night. Following incubation, washing was performed three times with ddH_2_O. To avoid unspecific binding, the plate was blocked by adding PBST containing 0.5% BSA and incubate for additional 30 min. After incubation the plate was washed three times with washing buffer (PBS with 0.05% Tween-20) and thereafter 2 μg of OPN was added to each well. The plate was incubated for 2 h at 37°C and washed three times in washing buffer. Primary antibody (rabbit-anti-OPN) was added to each well and incubated for 1 h at 37°C, followed by three times washing with washing buffer. Next, secondary antibody (HRP-conjugated goat-anti-rabbit) was added to each well and incubated for 1 h at 37°C followed by washing three times with washing buffer. ABTS buffer was added to each well and incubated for additional 30 min at room temperature to develop color and plate was read at 405 nm in an ELISA-reader.

### Bacteria and growth conditions

The *Streptococcus pneumoniae* strain TIGR4 is a clinical encapsulated isolate of serotype 4 sequenced by The Institute for Genomic Research TIGR (ATCC BAA-334; Rockville, MD). The strain was routinely grown in Todd-Hewitt (TH; Difco/Becton & Dickinson, Franklin Lakes, NJ) broth supplemented with 0.5% yeast extract (THY) liquid medium or on blood-agar plates. The *Pseudomonas aeruginosa* strain PA01, were grown in Todd-Hewitt (TH) medium or on agar plates in 5% CO_2_ and at 37°C.

### Bacterial killing assay

*S*. *pneumoniae* and *P*. *aeruginosa* were grown to mid-log phase in TH broth, washed, and diluted in incubation buffer (10 mM Tris-HCl, containing 5 mM glucose, pH 7.4). 50 μl of bacteria (2 x 10^6^ colony forming units (cfu)/ml) were incubated in the absence or presence of AMPs at various concentrations, (0.03–3 μM) for one hour at 37°C. Serial dilutions of the incubation mixtures were plated on blood agar plates or TH agar plates, followed by incubation at 37°C overnight whereafter the number of cfu was determined. In order to investigate the activity of the AMPs in the presence of OPN or the OPN-derived peptide VSS60, pre-incubations at 1:1 ratio were performed for 30 minutes prior to the bacterial killing assay.

### Scanning electron microscopy

For scanning electron microscopy (SEM), bacteria were incubated at 37°C for 1 hour in buffer alone, with OPN (3 μM), with AMPs (all at 3 μM), or with pre-incubated AMPs and OPN at equimolar concentrations. To verify bacterial killing, part of the sample was used for the bacterial killing assay described above. The samples were fixed in glutaraldehyde (0.5%), processed for SEM and examined in an XL 30 FEG scanning electron microscope (Philips, Eindhoven, The Netherlands).

### Muramidase-activity assay

The muramidase activity of lysozyme was tested by incubating lysozyme alone at 0.2 and 0.4 μM or pre-incubated mixture of lysozyme and OPN at equimolar concentrations for 1 hour at 37°C. After incubation the mixtures were tested for muramidase-activity exerted by lysozyme using EnzChek lysozyme assay kit (Molecular Probes, ThermoFisher Scientific, Göteborg, Sweden) as per the manufacturer’s instructions. The enzyme activity was measured as relative fluorescence units (RFU).

### Protease inhibition assay

Human neutrophil elastase (NE) (0.05 U/ml) was incubated either with SLPI alone or with a pre-incubated mixture of SLPI and OPN at equimolar concentration. The reaction mixture was tested for NE activity by using a chromogenic substrate (MeO-SucAAPVpNA) solution at a concentration of 15 mM (Sigma-Aldrich, St. Louis, MO). The absorbance was determined by spectrophotometric reading at 405 nm.

### Molecular modeling

Structural models of AMPs were prepared using Swiss-pdb Viewer version 4.1 (http://spdbv.vital-it.ch/disclaim.html) (PDB entries: 2BJJ, 1REX, 1KJ6, and 2LUT for lactoferrin, lysozyme, hBD-3, and midkine respectively).

### Statistical analyses

For statistical evaluation of more than two experimental groups, two-way ANOVA with Sidak’s multiple comparisons test was used and displayed as means ±SD. The statistical evaluations were performed using the GraphPad Prism software 6.0 (GraphPad Software, La Jolla, CA) with non-significant being *P*> 0.05 and the level of significance being *P ≤ 0.05, ** *P* ≤0.01, ***P ≤ 0.001, and ****P ≤ 0.0001.

## Results

### Co-localization of OPN and AMPs in COPD lung tissues

**Fig 1:**
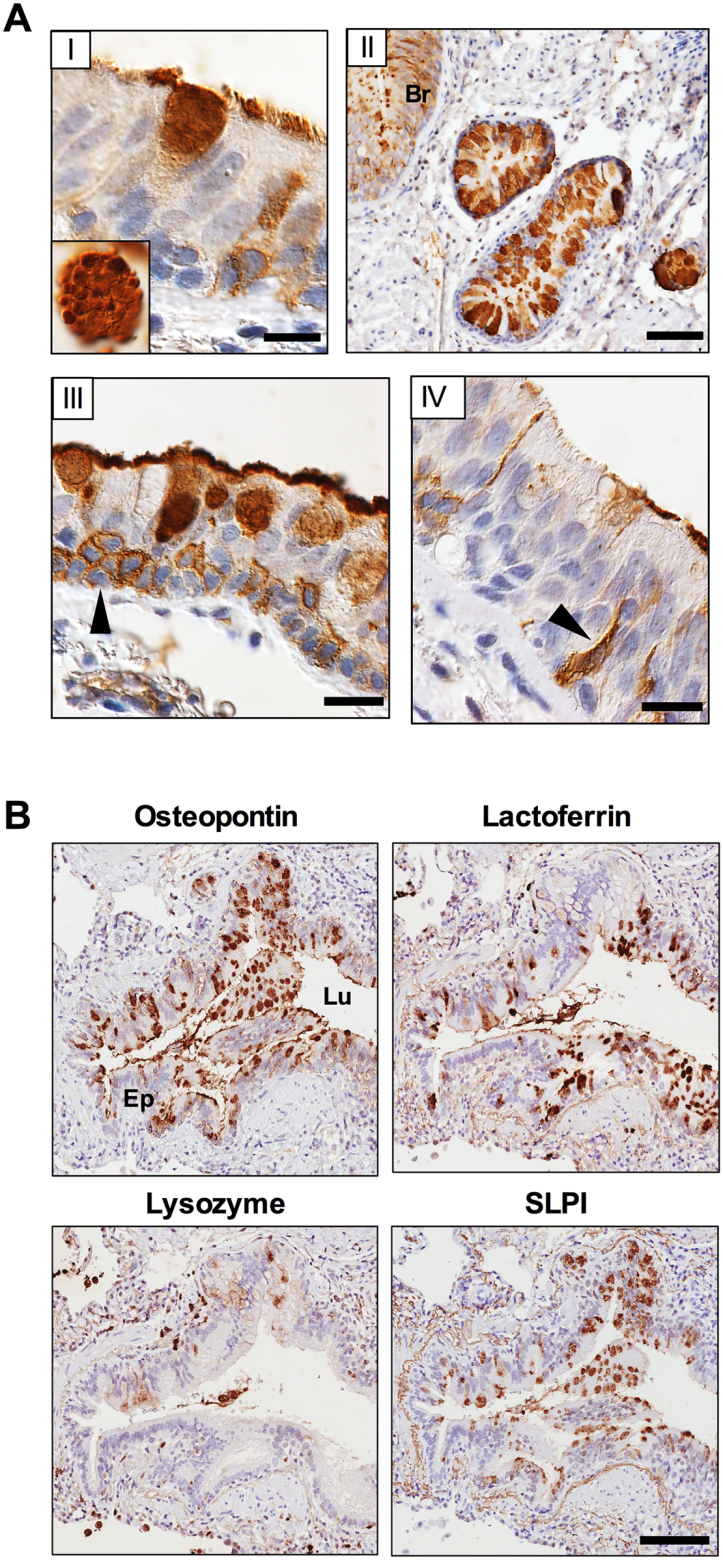
OPN expression and co-localization with antimicrobial proteins in COPD lung tissues. (A) Multiple cells in the bronchiolar epithelium expressed OPN (brown-colored DAB). (I) Goblet cells, the inset shows OPN expression in the goblet cell mucus. (II) Bronchial submucosal glands (Br = bronchial epithelium. (III) OPN expression in some basal cells (arrowhead). (IV) Flask-shaped cells (arrowhead). Primary antibodies from non-immunized animals resulted in loss of labeling (not shown). Scale bars: (I) = 15 μm; (II) = 70 μm; (III, IV) = 20 μm. (B) Immunohistochemical staining for OPN and the AMPs lactoferrin, lysozyme, and SLPI performed on parallel sections of lung tissue obtained from a patients with COPD (GOLD stage IV). Immunoreactivity is visualized by a brown-colored DAB staining in the bronchiolar epithelium and also in the airway lumen of COPD lungs. OPN, lactoferrin, and SLPI are all detected both in bronchiolar epithelium (Ep) and in cellular debris and mucus of the lumen (Lu) while lysozyme is present only in the lumen and to a lesser extent in the airway epithelium. The latter is explained by its preferential expression in the submucosal glands of large airways (not shown). Cell nuclei are counterstained with Mayer’s hematoxylin (blue stain). The scale bar in the right panel of the bottom figure is 100 μm.

Immunohistochemistry revealed that several types of cells in the small airway epithelium expressed OPN, including goblet cells, epithelial cells of submucosal glands, some basal cells, and flask-shaped cells (Fig 1A). Replacement of primary antibodies with IgG from non-immunized rabbits or goats respectively resulted in loss of labeling (not shown).

First, a possible co-localization of OPN with the most abundant airway AMPs lactoferrin, lysozyme, and SLPI was investigated [22]. Immunohistochemical staining was performed on thin parallel sections of lung tissue obtained from patients suffering from severe COPD (GOLD stage IV) (Fig 1B). Similarly to OPN, lactoferrin and SLPI were detected in the bronchiolar epithelium and in the lumen, providing strong evidence that OPN co-localizes with AMPs expressed in the airway epithelium. Lower amounts of lysozyme were detected in the lumen where neutrophils are the likely source but not in the bronchiolar epithelium, the latter explained by its expression mainly in serous submucosal glands of the large airways that were not included in the tissue investigated [23].

### Binding interactions between OPN and AMPs

**Fig 2:**
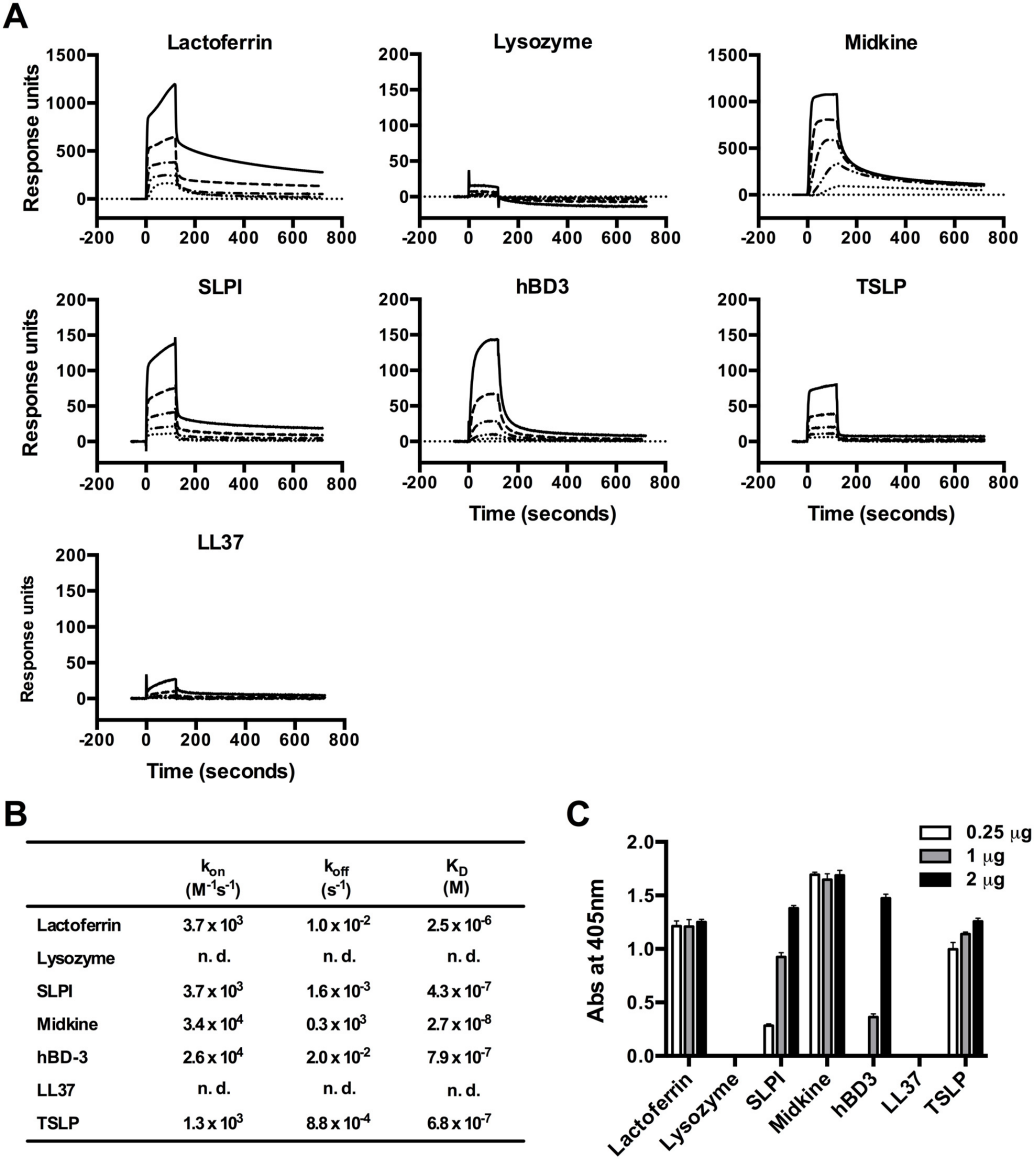
Binding of OPN to antimicrobial proteins. (A) Surface plasmon resonance (SPR) sensorgrams illustrating interactions between different AMPs (analyte) and immobilized OPN (ligand). The curves were obtained after injection of different concentration of the AMPs (31.25, 62.5, 125, 250, and 500 μM respectively) and analysis shows binding incidence with association and dissociation curves between lactoferrin, SLPI, midkine, hBD-3, TSLP, and OPN. No significant binding to OPN was observed for lysozyme and LL-37. (B) The association rate constants (k_on_), the dissociation rate constants (k_off_), and the equilibrium binding constants (K_D_) as calculated from the SPR. The association kinetics between OPN and neither lysozyme nor LL-37 could be determined (n. d.). (C) ELISA-based analysis of interaction between AMPs and OPN to confirm the binding pattern obtained using SPR. A 96 well plate was coated with 0.25, 1 and 2 μg of AMPs. Thereafter, the wells were incubated with 2 μg OPN, washed, followed by detection of bound OPN with antibodies. The histogram represents mean absorbance at 405 nm with standard deviation for each AMP. The incubations were performed in triplicates.

Possible binding interactions between OPN and AMPs (i.e. lactoferrin, lysozyme, SLPI, midkine, hBD-3, LL-37, and TSLP) were investigated using surface plasmon resonance (SPR) analysis. To determine the association and dissociation kinetics, varying concentrations of the individual AMPs were injected over OPN, the latter immobilized on a biosensor chip (Fig 2A). A dose-dependent binding to OPN was observed with lactoferrin, SLPI, midkine, hBD-3 and TSLP, while lysozyme and LL-37 showed very low or no binding. The association rate constants (k_on_), the dissociation rate constants (k_off_), and the equilibrium binding constants (K_D_) were calculated from the SPR (Fig 2B). However, the association kinetics between OPN and neither lysozyme nor LL-37 could be determined. The binding interactions were further confirmed using an ELISA-based binding assay (Fig 2C). In this assay, the AMPs were coated in a 96 well plate followed by incubation with OPN. Subsequently, detection of bound OPN was performed with an anti-OPN antibody. Consistent with the SPR analysis, lactoferrin, SLPI, midkine, hBD-3, and TSLP all displayed strong binding to OPN but neither lysozyme nor LL-37 exhibited binding affinity. Overall, the SPR data correlates well with ELISA data, indicating that OPN preferentially binds the AMPs that are highly cationic.

### Bactericidal properties of AMPs in the presence of OPN

The antimicrobial activity of AMPs was tested against *S*. *pneumoniae* and *P*. *aeruginosa*, both pathogens that are frequently seen in COPD patients [3, 6]. Bacteria were incubated with different concentrations of AMPs for one hour at 37°C whereafter viable counts were performed to determine bactericidal activity (S1 Fig). The AMPs investigated all showed high bactericidal activity against *S*. *pneumoniae* at a concentration of 0.3 μM with the exception of lactoferrin where a concentration of 3 μM was required to reach complete killing. In the case of *P*. *aeruginosa*, LL-37 displayed a somewhat lower bactericidal activity compared with the other AMPs investigated.

**Fig 3:**
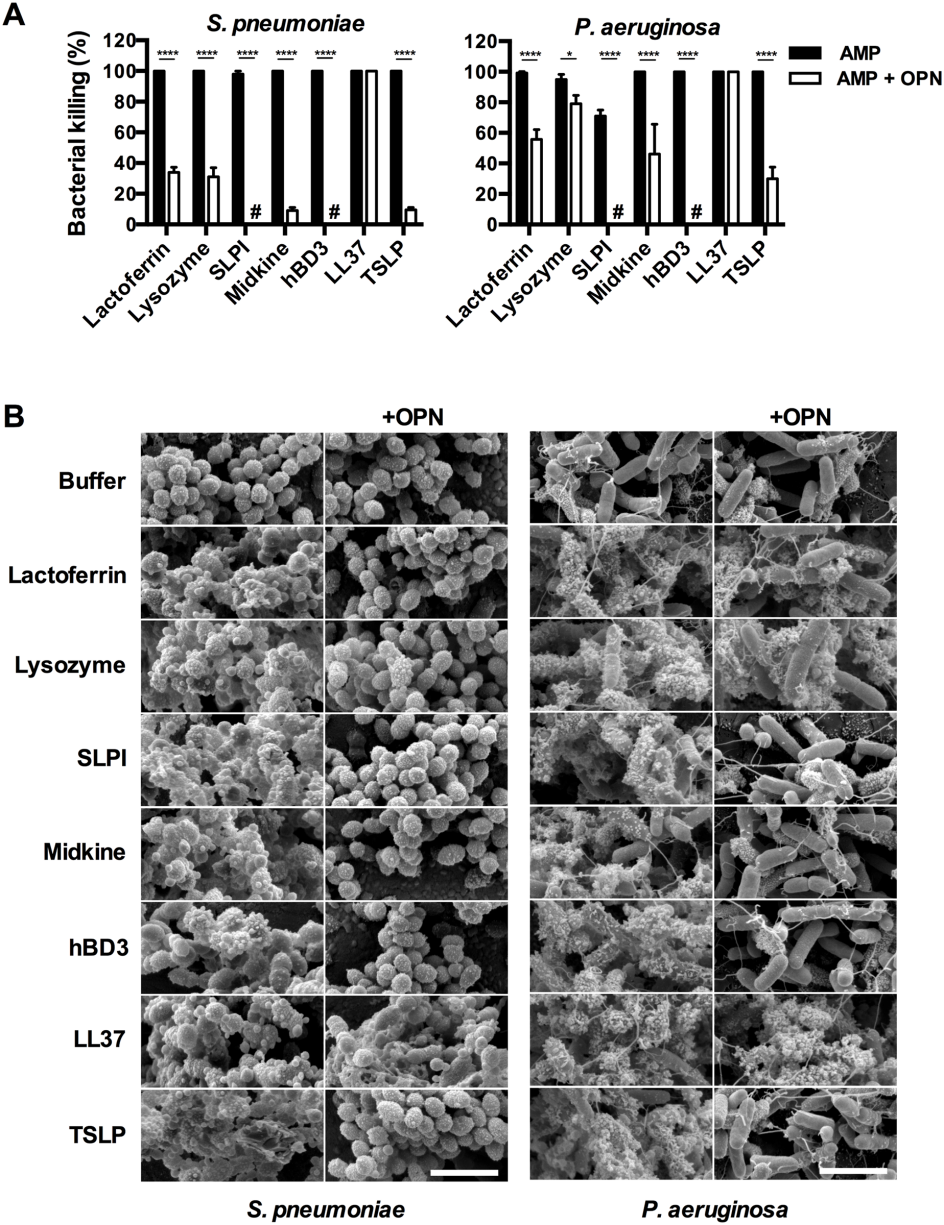
OPN impairs the bactericidal activity of AMPs. (A) Interference of OPN with the bactericidal activity of AMPs was investigated using viable counts with *S*. *pneumoniae* and *P*. *aeruginosa*. Bacteria were grown to mid-logarithmic phase and incubated with AMPs alone (3 μM) or AMPs pre-incubated with OPN at ratio of 1:1 for one hour at 37°C. OPN caused inhibition of the bactericidal activity of all AMPs investigated except for LL-37. The histograms represent mean and standard deviation from three separate experiments. Two-way ANOVA with Sidak’s multiple comparisons test was used for statistical analysis. *P≤0.05, and ****P ≤ 0.0001. (B) Scanning electron microscopy images shows the morphology of bacteria after incubation with AMPs alone or AMPs pre-incubated with OPN (1:1). The AMPs alone permeabilized the membrane and caused leakage of intracellular contents indicating killing of the bacteria, which was confirmed in a parallel viable counts assay. Upon co-incubation of AMPs and OPN, the bacteria remained intact, except in the case of LL-37 and to some extent in the cases of *P*. *aeruginosa* (OPN with lactoferrin, lysozyme, and midkine). The scale bars in bottom figures in the right panels are 5 μm.

To determine whether an interaction with OPN inhibits the antibacterial activity, the AMPs (3 μM) were pre-incubated with equimolar concentration of OPN and the bactericidal activity against *S*. *pneumoniae* and *P*. *aeruginosa* respectively was determined using viable counts (Fig 3A). All AMPs except LL-37 showed a strongly reduced bactericidal activity against *S*. *pneumoniae* in the presence of OPN (Fig 3A). A strong reduction was also seen when OPN was co-incubated with SLPI and hBD-3 respectively against *P*. *aeruginosa* (Fig 3A). However, when incubated with *P*. *aeruginosa*, the inhibition of antibacterial activity by OPN was less pronounced for lactoferrin, lysozyme, midkine, and TSLP and again not present at all with LL-37.

To visualize the morphology of bacteria during the conditions described above, scanning electron microscope (SEM) was used (Fig 3B). Incubating *S*. *pneumoniae* and *P*. *aeruginosa* with the AMPs alone resulted in a severely disturbed morphology, indicating permeabilization of the cell membrane and leakage of intracellular contents suggesting killing of the bacteria, which was confirmed in parallel viable counts experiments. In contrast, *S*. *pneumoniae* incubated with AMPs in the presence of OPN showed less leakage and most of the bacteria remained intact indicating inhibition of bactericidal properties of AMPs by OPN, except in the case of LL-37. For *P*. *aeruginosa*, bacteria remained intact when OPN was co-incubated with SLPI or hBD-3, to a lesser extent in the case of OPN combined with lactoferrin, lysozyme, midkine, or SLP and not at all when OPN was co-incubate with LL-37. The data clearly reveal dissimilarity in the bactericidal activities of AMPs in presence of OPN indicating the possible role of OPN as modulator of host defense functions where LL-37 stands out by not being affected by OPN.

### Inhibition of AMP activity by a fragment of OPN generated by elastase of *P*. *aeruginosa*

**Fig 4:**
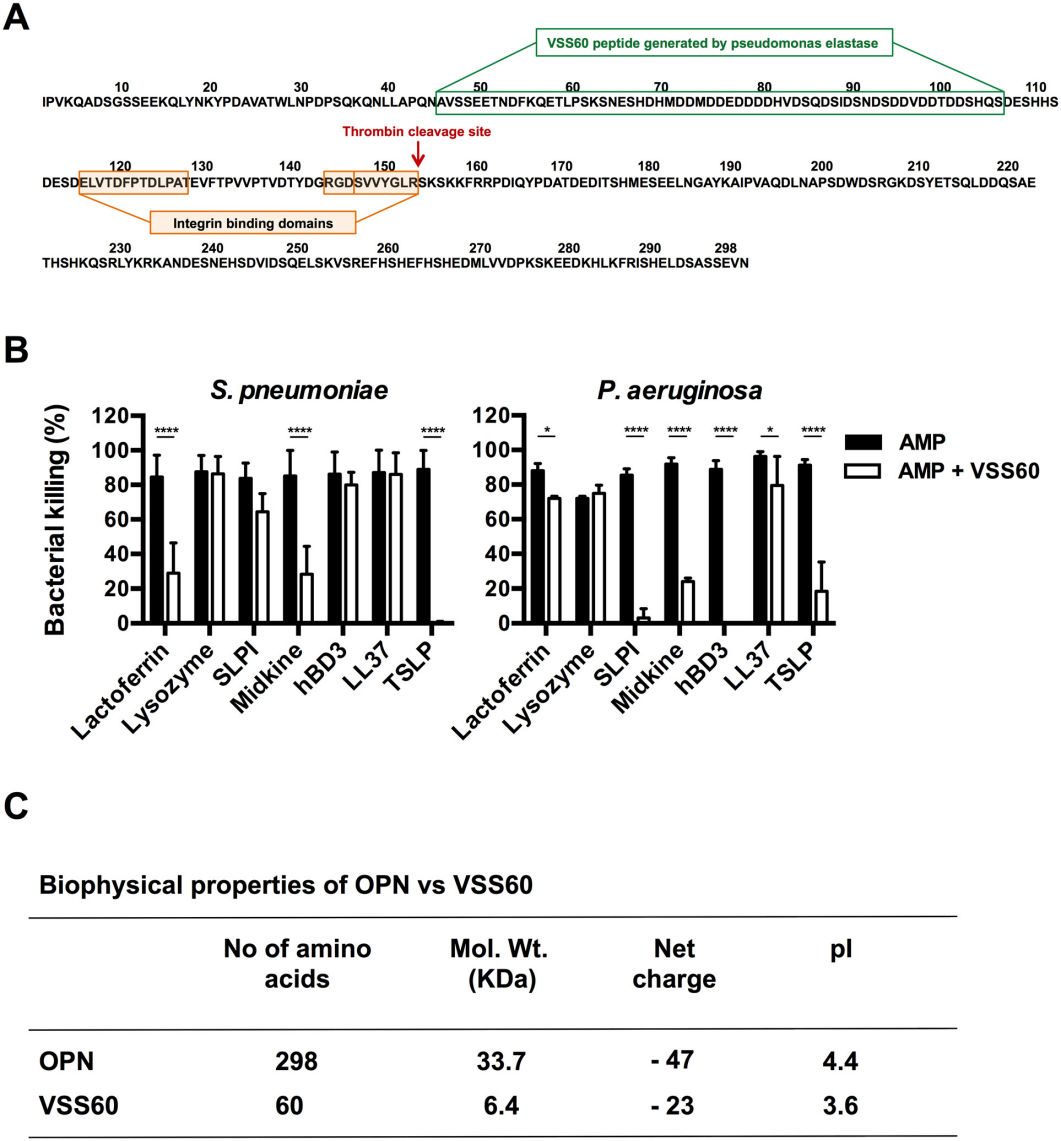
Impairment of the bactericidal activity of AMPs from the OPN-fragment VSS60 that is generated by elastase of *P*. *aeruginosa*. (A) Amino acid sequence of full length OPN and the VSS60-peptide (highlighted in green) which is generated by elastase of *P*. *aeruginosa*. The full-length OPN has an RGD-motif providing a binding site for integrins (highlighted in orange). (B) To investigate whether the VSS60-peptide retains the AMP-neutralizing properties of full-length OPN, *P*. *aeruginosa* were incubated in either buffer alone, with AMPs (3 μM), or AMPs pre-incubated with VSS60 peptide at a molecular ratio of 1:1 before addition to bacteria and incubated for one hour at 37°C. The antimicrobial activity was determined using viable counts. The VSS60-peptide reduced the bactericidal activity of SLPI, hBD-3, and TSLP. A similar but lower inhibitory activity compare with full length OPN. The histograms represent mean and standard deviation from three separate experiments. Two-way ANOVA with Sidak’s multiple comparisons test was used for statistical analysis. *P≤0.05, and ****P ≤ 0.0001. (C) Biophysical properties of OPN and peptide VSS60 derived from OPN showing number of amino acids, molecular weight, net charge and pI.

*P*. *aeruginosa* releases an elastase that generates a 60 amino acid peptide (VSS60) from full-length OPN (Fig 4A) [24]. To investigate whether this peptide retains properties that inhibit AMPs, viable counts was performed with *P*. *aeruginosa* and lactoferrin, lysozyme, SLPI, midkine, hBD-3, LL-37, and TSLP respectively (all at 3 μM) either in presence or absence of equimolar concentration of the VSS60-peptide (Fig 4B). Similarly to the full-length protein, the OPN-fragment showed the most pronounced inhibition of the bactericidal activity of SLPI, hBD-3, and TSLP. No inhibitory activity was seen for the other AMPs investigated. The properties of full-length OPN and VSS60-peptide are shown in Fig 4C.

### Functional activity of lysozyme and SLPI in the presence of OPN

**Fig 5:**
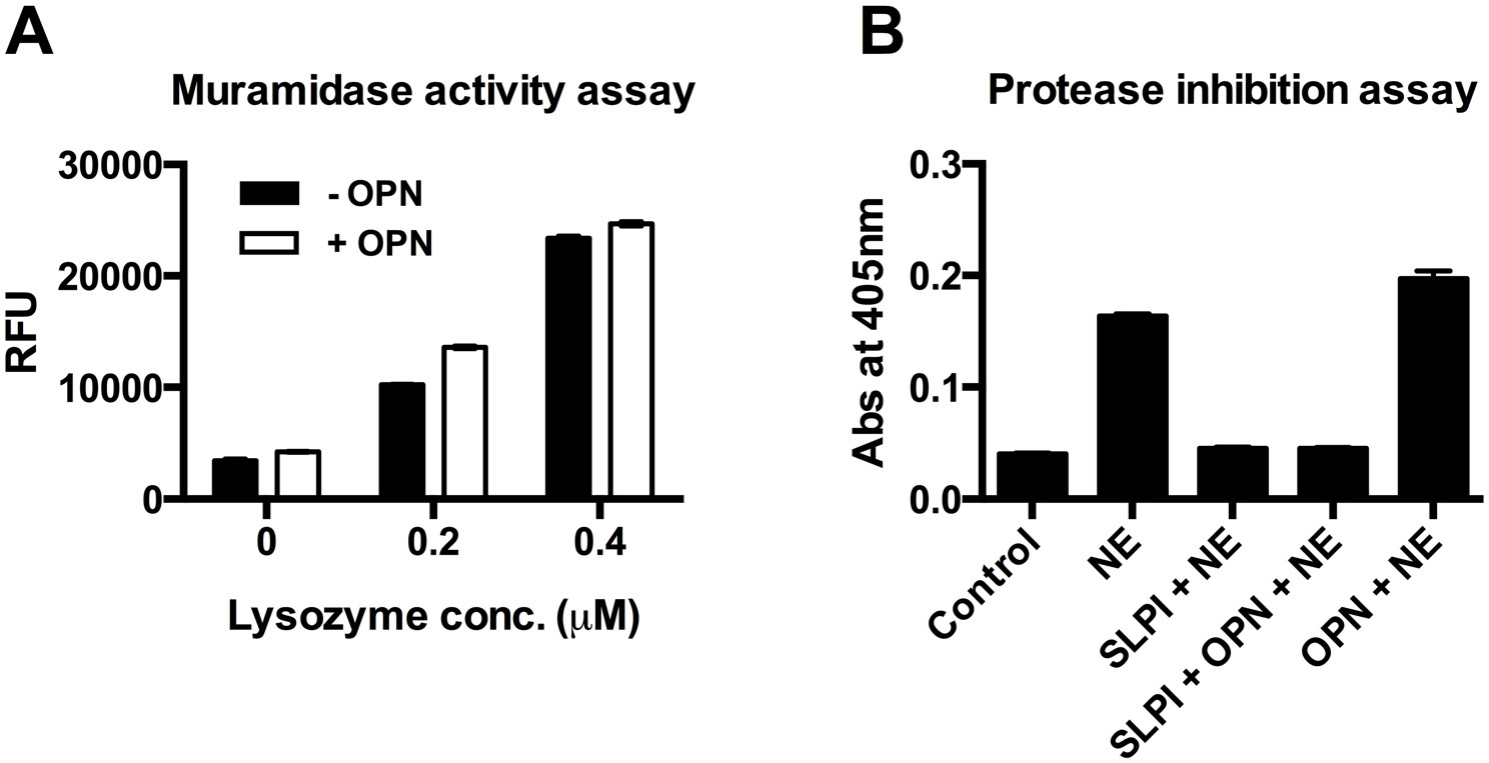
OPN does not influence the muramidase and protease inhibitory functions of lysozyme and SLPI respectively. (A) Effect of OPN on muramidase activity of lysozyme. Lysozyme was pre-incubated with or without OPN at equimolar concentrations and fluorogenic substrate was added to the mixture and incubated for 1 h at 37°C. The lysozyme activity was determined by the development of fluorescence, which is represented as relative fluorescence units (RFU). (B) Neutrophil elastase inhibitory property of SLPI was investigated in presence of OPN. Equimolar concentrations of OPN and SLPI were pre-incubated for 1 h at 37°C. This mixture was incubated with human neutrophil elastase (NE) (0.05 U/ml) for 20 min at RT. The NE activity was determined by a chromogenic substrate solution by recording the absorbance at 405 nm. The above experiments suggest that OPN cannot influence the enzymatic activities of lysozyme and SLPI.

In addition to the inhibitory properties of OPN with respect to bactericidal activity of AMPs, possible interference from OPN on the muramidase and protease-inhibitory activities of lysozyme and SLPI respectively were investigated. At equimolar concentrations, the muramidase activity of lysozyme and the protease inhibitory function of SLPI were not affected (Fig 5). These observations suggest a role for OPN as a modulator of the antimicrobial functions of lysozyme and, in particular SLPI.

## Discussion

In this study, we show that OPN binds and impair the bactericidal activity of several AMPs that are expressed in the airways during COPD. The finding can, at least in part, explain the increased vulnerability to acquire bacterial infections in COPD.

Lysozyme and lactoferrin are the most abundant AMPs in airway lavage fluid and are found at approximately 10 μg/mL while SLPI is found at 0.1–2 μg/mL [22, 25, 26]. The other AMPs investigated in this study may also reach high levels in the microenvironment. To our knowledge, the key importance of a single AMP has not been shown except for impaired bacterial host defense in mice deficient of CRAMP, a murine orthologue of LL-37, and lysozyme [27, 28]. Thus, there is likely a high redundancy in this class of host defense molecules. In addition, the paired combinations of lysozyme-lactoferrin, lysozyme-SLPI, and lactoferrin-SLPI have synergistic activities and the triple combination of lysozyme, lactoferrin, and SLPI show even greater synergy [12]. Do then OPN reach significant levels in the airways during COPD to allow interactions with these AMPs? In sputum of COPD patients, OPN was detected at 0.6–6.2 μg/mL [21]. However, it seems that OPN have a preference for neutralizing the activity of SLPI and hBD-3.

What is the nature of the interaction between OPN and AMPs? As deduced from the SPR experiments, the interactions are mainly electrostatic. This is also suggested by the highly anionic nature of OPN with a pI of 4.1 on an amino acid level where post-translational modifications (i.e. addition of sialic acid-containing carbohydrates and phosphate groups) further add anionic charge to the molecule. The AMPs on the other hand have a high cationic net charge (S2 Fig). The importance of the anionic charge of OPN is also demonstrated by the lower neutralizing activity executed by the OPN-fragment VSS60, being less anionic than full-length OPN.

The lack of binding between OPN and LL-37 is surprising but is supported by the absence of interference in the bactericidal assays. One explanation could be the distribution of cationic amino acids and conformation, resulting in that charge interactions between the molecules do not occur. A paradoxical finding is that OPN impairs the bactericidal activity of lysozyme, in particular in the case of *S*. *pneumoniae*, but show no or very low interactions in the binding assays. One explanation could be that OPN binds to the surface of bacteria, thereby providing protection as previously shown [29]. The binding of OPN to the bacterial surface could provide a protective shield, neutralizing the activity of lysozyme.

An animal model of pneumococcal airway infections supports that OPN impairs airway host defense against this bacterium [29]. In this study, OPN-binding to the surface of *S*. *pneumoniae* was demonstrated, increasing the resistance of the bacteria to hypotonic lysis. However, a possible resistance to AMPs was not investigated in this study.

Lysozyme has muramidase activity that can degrade the peptidoglycans in the cell wall of bacteria, thereby acting synergistic with the bactericidal activity of beta-defensins [30]. However, we found no inhibition of the muramidase activity by OPN, suggesting that binding does not interfere with the catalytic site. High and uncontrolled protease-activity, in particular by serine-proteases released by neutrophils, is a characteristic pathophysiologic feature of COPD. Thus, protease-inhibition as executed by SLPI is important and we found no decrease in protease-inhibition upon incubation with SLPI.

Why is the function of the highly up-regulated OPN-expression in COPD? One can speculate that OPN is part of the prolonged and dysregulated inflammation seen in COPD where pro- and anti-inflammatory mechanisms are not functionally coordinated. Thus, OPN could be part of an attempt to achieve resolution of inflammation, which is supported by upregulation of its gene expression by the anti-inflammatory cytokine IL-10 [31, 32]. However, the gene-regulation in bronchial epithelial cells during COPD remains to be investigated.

Taken together, during the high and dysregulated state of inflammation seen in COPD, the elevated levels of OPN can impair the activity of AMPs, promoting vulnerability to acquire bacterial infections. OPN itself or its gene-regulation are interesting targets for novel therapeutic approaches in this disease.

## Supporting Information

S1 Fig Antimicrobial activity of AMPs. The bactericidal activity of AMPs (i.e. lactoferrin, lysozyme, SLPI, midkine hBD-3, LL-37 and TSLP) was investigated against *S*. *pneumoniae* and *P*. *aeruginosa*. Bacteria were grown to mid-logarithmic phase and incubated with varying concentrations of the AMPs (0.03, 0.1, 0.3, 1, and 3 μM respectively) for 1 h at 37°C. The antimicrobial activity was determined by plating serial dilution of bacteria on agar plates and number of cfu was counted after overnight incubation. The AMPs investigated showed dose dependent bactericidal activity and 100% bacterial killing was achieved in all cases at 3 μM concentration. The histogram represents mean and standard deviation from three separate experiments.
(TIFF)

S2 Fig Structure modeling and biophysical properties of the AMPs investigated. (A) Three dimensional molecular models of lactoferrin, lysozyme, midkine and hBD-3. The positively charged amino acids arginines, and lysines are highlighted in blue. (B) Biophysical properties of AMPs and OPN showing number of aminoacids, molecular weight, net charge and pI.
(TIFF)

S1 Table Clinical characteristics of patients where lung-tissue was obtained for immunohistochemistry. (PDF)
