# Supplementary figures and images for: Osteopontin That Is Elevated in the Airways during COPD Impairs the Antibacterial Activity of Common Innate Antibiotics
Source: PLoS One. 2016 Jan 5;11(1):e0146192. doi: 10.1371/journal.pone.0146192 (PMC4712133; doi:10.1371/journal.pone.0146192)

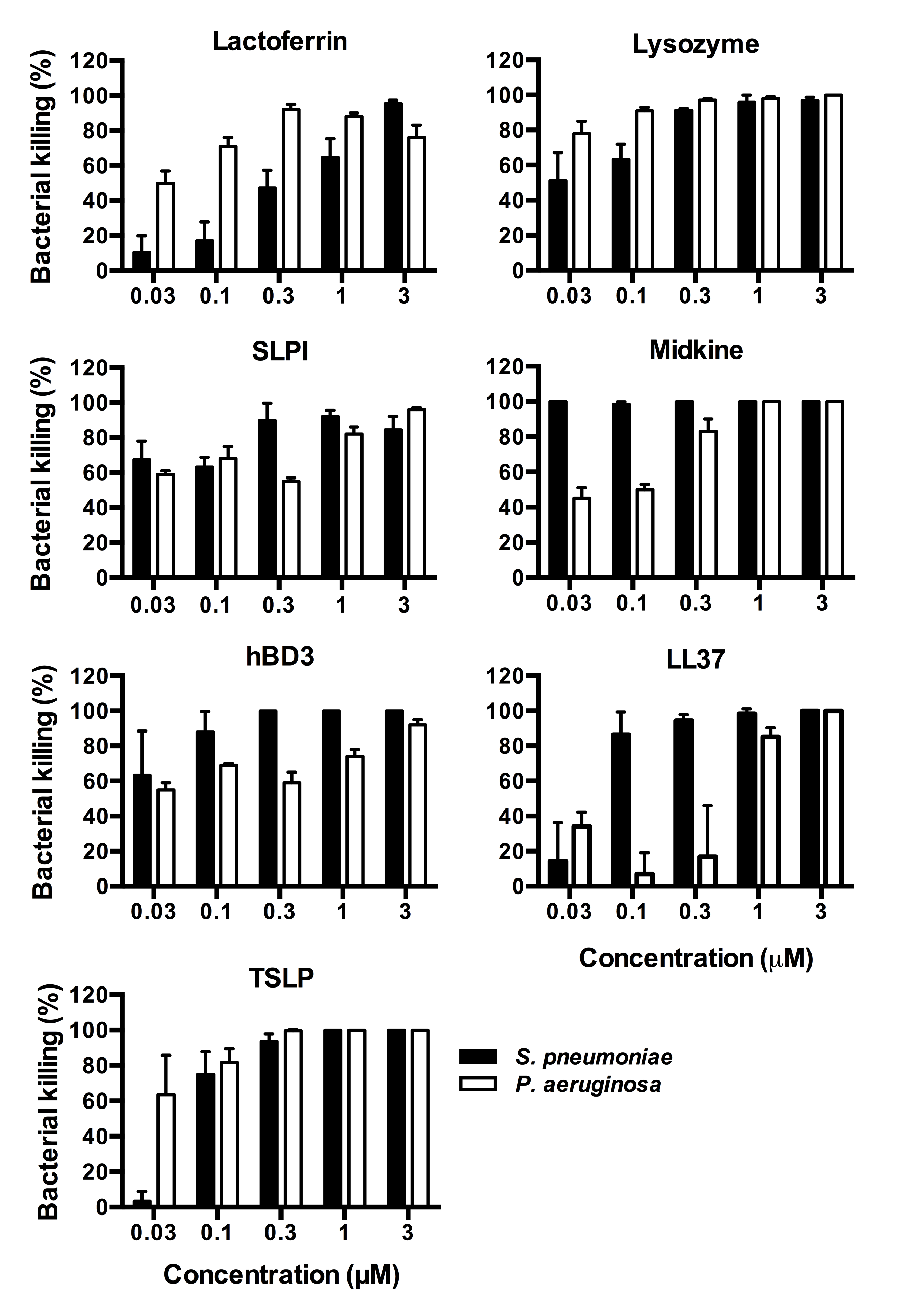

Supplement: S1 Fig — The bactericidal activity of AMPs (i.e. lactoferrin, lysozyme, SLPI, midkine hBD-3, LL-37 and TSLP) was investigated against S. pneumoniae and P. aeruginosa. Bacteria were grown to mid-logarithmic phase and incubated with varying concentrations of the AMPs (0.03, 0.1, 0.3, 1, and 3 μM respectively) for 1 h at 37°C. The antimicrobial activity was determined by plating serial dilution of bacteria on agar plates and number of cfu was counted after overnight incubation. The AMPs investigated showed dose dependent bactericidal activity and 100% bacterial killing was achieved in all cases at 3 μM concentration. The histogram represents mean and standard deviation from three separate experiments. (TIFF) [file pone.0146192.s001.tiff]

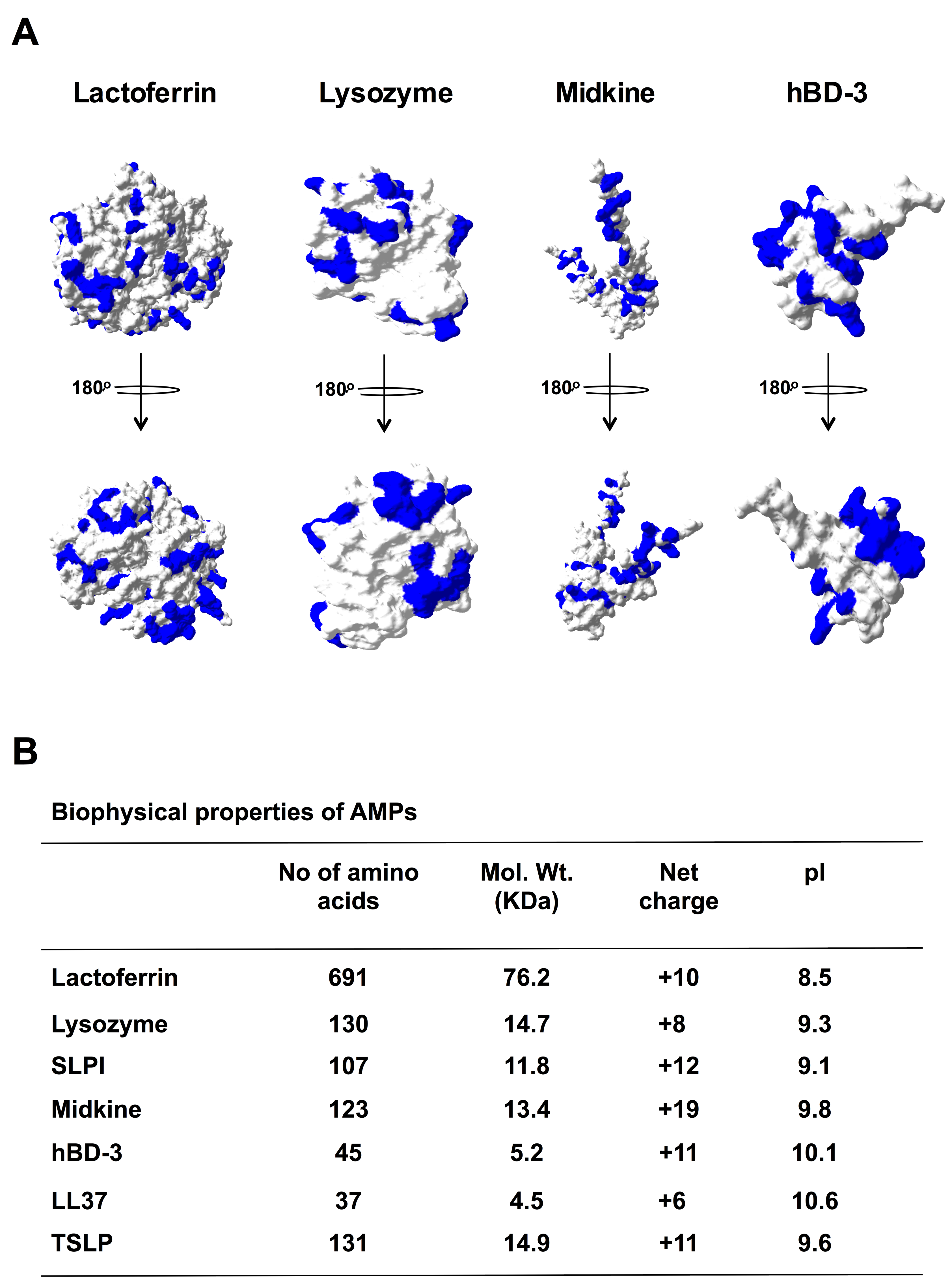

Supplement: S2 Fig — (A) Three dimensional molecular models of lactoferrin, lysozyme, midkine and hBD-3. The positively charged amino acids arginines, and lysines are highlighted in blue. (B) Biophysical properties of AMPs and OPN showing number of aminoacids, molecular weight, net charge and pI. (TIFF) [file pone.0146192.s002.tiff]
