# Supplementary material for: Osteopontin That Is Elevated in the Airways during COPD Impairs the Antibacterial Activity of Common Innate Antibiotics
Source: PLoS One. 2016 Jan 5;11(1):e0146192. doi: 10.1371/journal.pone.0146192 (PMC4712133; doi:10.1371/journal.pone.0146192)
Supplement: S1 Table — (PDF) [file pone.0146192.s003.pdf]

S1 Table:

Clinical characteristics of patients where lung-tissue was obtained for immunohistochemistry.

	**Patient 1**	**Patient 2**
**GOLD stage**	**IV**	**IV**
Gender	F	F
Age (years)	53	65
Pack-years	32	30
Ex-smokers	Yes	Yes
FEV_1	0.6	0.5
FEV_1, % of predicted	20	24
FEV_1/FVC, %	32	32
Inhaled β_2 agonists	No	Yes
Inhaled anticholinergics	Yes	Yes
Inhaled short acting β_2 agonist plus anticholinergics	Yes	No
Corticosteroids (inhaled)	No	Yes
Inhaled long-acting β_2 agonist plus corticosteroids (yes)	Yes	No

COPD, chronic obstructive pulmonary disease; FEV_1, forced expiratory volume (L) in one second; FVC, forced vital capacity; GOLD, Global Initiative for Chronic Obstructive Lung Disease.
